# ROBOTIC TRANS-ABDOMINAL PREPERITONEAL APPROACH (TAPP) APPROACH FOR LATERAL INCISIONAL HERNIAS

**DOI:** 10.1590/0102-672020210002e1599

**Published:** 2021-10-18

**Authors:** Ana T Garcia CABRERA, Diego Laurentino LIMA, Xavier PEREIRA, Leandro Totti CAVAZZOLA, Flavio MALCHER

**Affiliations:** 1 Montefiore Medical Center, Department of Surgery, The Bronx, New York, USA; 2 Department of Surgery, Federal University of Rio Grande do Sul, Porto Alegre, RS, Brazil

**Keywords:** Robotic surgical procedures, Incisional hernia, Procedimentos cirúrgicos robóticos, Hérnia incisional

## Abstract

**Background:**

Lateral incisional hernias arise between the linea alba and the posterior paraspinal muscles. Anatomical boundaries contain various topographic variations, such as multiple nearby bony structures and paucity of aponeurotic tissue that make it particularly challenging to repair.

**Aim:**

To describe a robotic assisted surgical technique for incisional lumbar hernia repair.

**Methods:**

Retrospective data was collected from four patients who underwent robotic-assisted repair of their lumbar hernias after open nephrectomies.

**Results:**

Age ranged from 41-53 y. Two patients had right sided flank hernias while the other two on the left. One patient had a recurrent hernia on the left side. The patients were placed in lateral decubitus position contralateral to the hernia defect side. A trans-abdominal preperitoneal approach was used in all cases. Each case was accomplished with two 8 mm robotic ports, a 12 mm periumbilical port, and a 5 mm assistance port that allowed docking on the ipsilateral hernia side. The hernias were identified, a preperitoneal plane was created, and the hernia sac completely dissected allowing for complete visualization of the defect. All defects were primarily closed. Polypropylene or ProGrip^TM^ mesh was applied with at least 5 cm overlap and secured using either #0 Vicryl^®^ transfacial sutures, Evicel^®^ or a combination of both. The peritoneal space was closed with running suture and the ports were removed and closed. The average surgical length was 4 hr. The post-operative length of stay ranged from 0-2 days.

**Conclusion:**

The robotics platform may provide unique advantages in the repair of lateral incisional hernias and represents a safe, feasible and effective minimally invasive approach for the correction of lateral incisional hernias.

## RESUMO

**Racional:**

As hérnias incisionais laterais surgem entre a linha alba e os músculos paravertebrais posteriores. Os limites anatômicos contêm várias variações topográficas, como várias estruturas ósseas próximas e escassez de tecido aponeurótico que tornam o reparo particularmente difícil *.*

**Objetivo:**

Descrever uma técnica assistida por robótica para o reparo de hérnia lombar incisional.

**Métodos:**

Dados foram coletados retrospectivamente de quatro pacientes que foram submetidos ao reparo de hérnia lombar após nefrectomias abertas por técnica robótica. Os pacientes tinham entre 41-53 anos de idade. Dois possuíam hérnia no flanco direito e os outros dois no flanco esquerdo.

**Resultados:**

Os pacientes foram colocados em posição de decúbito lateral contralateral ao lado do defeito. Abordagem pré-peritoneal transabdominal foi realizada em todos os casos. Cada procedimento foi realizado com dois trocárteres robóticos de 8 mm, um periumbilical de 12 mm e um auxiliar de 5 mm, permitindo docking ipsilateral ao lado da hérnia. As hérnias foram identificadas, plano pré-peritoneal foi criado e o saco herniário completamente dissecado, permitindo completa visualização do defeito. Todos os defeitos foram fechados primariamente com fio de sutura 0/1V-Loc. Tela de polipropileno ou ProGrip^TM^ foi usada com pelo menos 5 cm de overlap e fixada com sutura transfacial com Vicryl^®^ 0, Evicel^®^ ou combinação dos dois. O espaço pré-peritoneal foi fechado com sutura contínua e os trocárteres removidos. O tempo operatório médio foi de 4 h. O tempo de permanência hospitalar variou entre 0-2 dias.

**Conclusão:**

A plataforma robótica é capaz de providenciar vantagens únicas no reparo de hérnias incisionais laterais e representa abordagem minimamente invasiva segura, factível e eficaz para o reparo das hérnias laterais incisionais.

## INTRODUCTION

Lateral incisional hernias (LIH) arise between the linea alba and the posterior paraspinal muscles, between the iliac crest and the costal margin^5^. These anatomical boundaries contain various topographic variations, such as multiple nearby bony structures and paucity of aponeurotic tissue, that make LIH particularly challenging to repair. These constrains can make it difficult to achieve a wide overlap of mesh, prevent primary closure of defects, and provide adequate mesh fixation^4^ ^,^ ^5^. The European Hernia Society has proposed a classification for lateral incisional hernias according to their position (subcostal, flank, iliac, lumbar), recurrence, length and width^2^.

Most technical reports in the literature have focused on open approaches for the repair of LIH with varying degrees of success^11^ ^,^ ^12^ ^,^ ^13^. There is a paucity of data regarding minimally invasive techniques for the repair of this challenging surgical entity^3^ ^,^ ^6^ ^,^ ^7^ ^,^ ^8^ ^,^ ^9^ ^,^ ^13^ ^,^ ^15^ ^,^ ^16^. In fact, there are very few reports in the literature that focus on robotic repairs for LIH^1^ ^,^ ^5^ ^,^ ^17^ Furthermore, they contain a very limited number of patients with limited post-operative follow-up. 

We aim to outline an emerging, robotic-assisted, lateral incisional hernia repair technique and to report on the outcomes. 

## METHOD

This study was approved by the institutional ethic committee under number IRB # 2020-11160. It is a retrospective analysis of four patients who underwent robotic trans-abdominal preperitoneal approach (TAPP) repair of their lateral incisional hernias after open nephrectomies.

### Technique

The patients were a 53 year-old male patient with an incisional hernia in the right flank, a 41 year-old male with a recurrent left sided incisional hernia, a 77 year-old female with an incisional hernia on the left flank, and a 62 year-old male with a right flank incisional hernia (Table 1). All patients were operated with the Da Vinci Si robot system (Intuitive Surgical).

**TABLE 1:** Patient characteristics

Patient	Age/Gender	BMI	Comorbidities	Laterality	Surgical history	Presentation
1	53 M	31	None	Right	Open nephrectomy, 1 y/a	Pain, bulging
2	41 M	35	Renal CA, GERD, HLD	Left	Open nephrectomy 7 y/a, open hernia repair 5 y/a	Pain
3	77 F	36	Renal CA, HTN, GERD, HLD.	Left	Open nephrectomy 1 y/a	Bulging
4	62 M	28	None	Right	Open partial nephrectomy 12 y/a	Bulging

BMI=body mass index; M=male; F=female; y/a=year ago; CA=cancer; GERD=gastroesophageal reflux disease; HLD=hyperlipidemia; HTN=hypertension; n/a=not available

#### Positioning of the patient and establishing pneumoperitoneum

**FIGURE 1:**
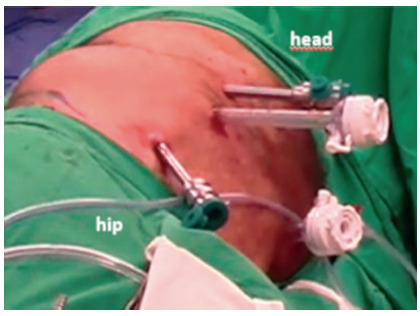
Port positioning

TAPP was used in all cases. The patients were placed in lateral decubitus position contralateral to the hernia defect side (Figure 1). The abdominal cavity was insufflated via a Veress needle to 15 mm Hg.

#### Trocar positioning

A 5 mm Optiview port was inserted through the ipsilateral subcostal area. One peri-umbilical 12 mm port and two 8 mm ipsilateral paramedian ports were placed and the robot was docked from the ipsilateral hernia side. The 4^th^ arm was used for retraction and some bed assist help (Figure 1).

#### Locating the defects and creating the pre-peritoneal plane

**FIGURE 2:**
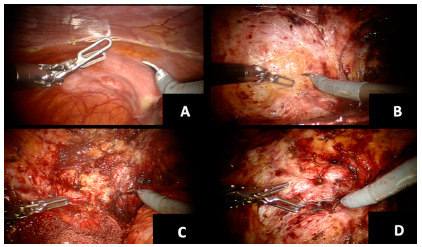
A) Peritoneal incision; B) pre-peritoneal plane dissection; C) retroperitoneal dissection; D) defect assessment.

The abdomen was explored until the lumbar herniating defects were encountered. Adhesiolysis and complete reduction of the hernia contents was performed as necessary. A peritoneal incision was made at least 5 cm medial to the edge of the defect (Figure 2A) and a large pre-peritoneal plane was created. (Figures 2B and C)

#### Dissection of the hernia sacs and defect closure

**FIGURE 3:**
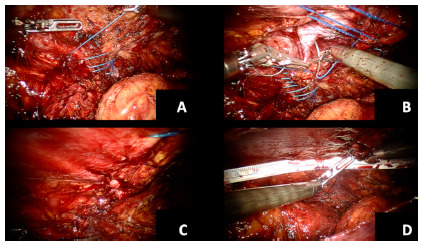
Defect management: A) initial closure; B) progressive closure; C) completed closure; D) final space measurement for mesh trimming.

The hernia sacs were completely dissected and reduced allowing for complete visualization of the hernia defect (Figure 2D). The respective defect sizes were 11x10 cm, 11x16 cm, 4x5 cm with a neighboring 1.5x2 cm defect, and 9x8 cm. The mean defect area was 99 cm^2^. All defects were primarily closed with V-Loc^TM^ (Medtronic MN, USA) sutures (Figures 3A, B, C). In the two cases with defects of over 9 cm, barbed sutures were used with progressive tensioning of the suture. Furthermore, reduction of pneumoperitoneum to 10 mm Hg was used to achieve an adequate tension closure of the hernia defects. On one occasion, a Progrip mesh was placed overlying the primary repair to reduce tension on the suture line. The large defect was closed under high tension and it was in close proximity to the ribs and anterior superior iliac spine. The concept was to use the large gripping surface of the mesh to reduce central tension on the closure followed by a bigger mesh to create an adequate overlap of the repair.

#### Positioning and fixation of the mesh

**FIGURE 4:**
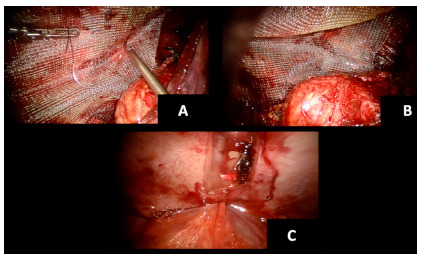
Mesh implantation: A) suture fixation of the mesh; B) final mesh positioning; C) peritoneal closure

Next, the preperitoneal space was measured (Figure 3D) and either Polypropyelene or ProGrip^TM^ mesh was trimmed to allow for at least 5 cm of overlap in all directions. The meshes were secured using either #2-0 Vicryl^®^ sutures, Evicel^®^ or a combination of both (Figures 4A, B). Transfacial sutures (two 0 Vicryl^®^ ties with a suture passer) were used in one case (11x16 cm defect), which helped with positioning and fixation of the mesh.

The peritoneal space was closed with running #2-0/3-0 Vicryl^®^/VLock and no residual defects were noted in the peritoneum (Figure 4C). The ports were removed, and the robot was undocked at this time. Average procedure length was 4 hr (Table 2).

**TABLE 2:** Intra-operative characteristics

Patient	Defect size (cm)	Closure	Mesh (cm)	Fixation	Peritoneal closure
1	11x16	0 V-Loc and 16x12 ProGrip mesh	24 x 20 medium weight PP	0 V-Loc, Vicryl transfacial, Evicel	3-0 V-Loc 2-0 Vicryl
2	4x5 1.5x2	0 V-Loc	15 x 15 ProGrip	--	3-0 V-Loc
3	9x8	0 V-Loc	20 x 15 ProGrip	Evicel	3-0 V-Loc
4	11x10	1 V-Loc	25 x 25 heavyweight PP	2-0 Vicryl	2-0 Vicryl

cm=centimeters; PP=polypropylene

## RESULTS

Post-operative length of stay ranged from 0-2 days. One of the patients was readmitted on the 2^nd^ post-operative day with a clinically symptomatic seroma and managed conservatively with pain control and observation. At 90 days, none of the patients developed recurrence or bulging at the surgical site and all had complete return to full physical activity (Table 3).

**TABLE 3:** Post-operative results

Patient	Duration (hr)	LOS (days)	Post-operative complication	Follow-up (months)
1	6.5	1	Seroma	2
2	3	0	None	4
3	2.5	0	None	1
4	4	2	None	24

hr=hour

## DISCUSSION

### Introduction to the problem

LIH remain a rare but challenging problem to most general surgeons. The unfamiliar anatomy, the constraints of the lateral abdominal wall, and the lack of an established surgical technique makes this surgical entity particularly difficult to treat^5^. As a result, there is a paucity of literature discussing repair techniques, the optimal surgical modality, and the outcomes after repair. This is especially true in minimally invasive approaches to LIH repairs^7^ ^-^ ^15^.

### Mini review of the literature

Renard et al^13^, Pezeshk et al^12^, and Patel et al^11^ published some of the earliest reports of open LIH. These studies are frequently cited and their results are often used as a comparison group in more recent reports discussing minimally invasive approaches to LIH. Renard et al^13^ evaluated 31 patients where 45% had recurrent lateral incisional hernias. The mesh was positioned totally extraperitoneal in 65%. His recurrence rate was 6.5% and 9.7% had chronic pain^13^. Pezeshk et al^12^ evaluated 29 patients who were submitted to open LIH and with a follow-up of 21.2 months. Only one patient had a recurrence^5^ ^,^ ^12^. Patel et al^11^ published his results of open LIH in 61 patients^11^. They used the European Hernia System Classification to describe their cases: 14 subcostal, 33 flank, 11 iliac and 3 lumbar hernias. Retromuscular, interparietal and preperitoneal repairs were mainly performed; 11.5% of the patients had recurrence of the hernia.^11^ Veyrie et al^16^ evaluated 61 patients undergoing laparoscopic LIH repair by the retro-muscular approach with a polyester standard prosthesis. There were 14 subcostal hernias, 12 flank hernias and 35 in the iliac fossa. The recurrence rate was 4.9% (n=3) and the median follow-up was 47 months (1-125). Sun et al^15^ performed a laparoscopic trans-abdominal partial extra-peritoneal (TAPE) repair in 14 patients, 13 of which had lateral incisional hernias and one after trauma. In this retrospective study there were no post-operative complications such as seroma, hematoma, wound infection, recurrence or bulging during the follow-up (median of 33 months). Edwards et al^2^ performed a retrospective study with 27 patients that were subjected to a laparoscopic transperitoneal flank incisional hernia repair. Average defect size was 188 cm^2^ and average mesh size of 650 cm^2^. Mean length of stay was 3.1 days and mean follow-up was 3.6 months (1-10). During this time, three patients had chronic pain and there was no recurrence. Shekarriz et al^14^ presented only three laparoscopic cases of LIH with no complications and a mean follow-up of 12 months. Lal et al^6^, with 25 patients, showed that the laparoscopic approach was as challenging as the open technique, feasible however with higher rates of complications and recurrence. Ferrarese et al^3^ analyzed 78 patients retrospectively and compared the defect locations.

There are only three studies in the literature showing robotic LIH^1^ ^,^ ^5^ ^,^ ^17^. Kudsi et al^5^ showed the robotic hernia repair in 26 patients with lateral incisional hernias. TAPP was performed in only eight patients. The technique consisted of closing the defect, deploying the mesh and closing the peritoneal flap with a barbed absorbable suture. There was no conversion to open or laparoscopic, with only one hybrid intraperitoneal onlay mesh procedure. There were no significant differences in the results between the groups. Two patients in the TAPP group developed a seroma. There were no postoperative infections. Fifteen out of 26 patients were discharged in the same day of the procedure and the mean LOS was 0.65 days. In a similar study, Di Giuseppe et al^1^ demonstrated a robotic approach in seven patients. In this study, the median hernia defect was 2.5 cm with a median operative time of 137 min. They had no intraoperative complications and a six months follow-up showed no recurrence or chronic pain. Wijerathne et al^17^ showed a tailored approach for lateral ventral hernia repairs in 22 patients. However, only three had robotic repairs (rTAPP). Four had laparoscopic TAPP, another four eTEP repair, nine were repaired with laparoscopic intraperitoneal on-lay mesh technique and closure of the defect and two with TAPE approach. Four patients developed seroma and no further complications were noted during a minimum of 12 months follow-up.

### Our conclusions and tips

In our report, we highlight four cases of successful treatment of LIH using a robotic-assisted approach. All patients had an incisional hernia from previous open nephrectomy and underwent successful TAPP repair of LIH. Laterality was equally distributed with a 3:1 predominance of male to female subjects. Mean age was 58 years old, all patients had an elevated BMI with a mean of 28.5. There were no conversions to open procedure. The average defect size was 76 cm^2^ with an average mesh size of 408 cm^2^. One patient was readmitted to the hospital with a symptomatic seroma that was successfully treated with supportive management.

Appropriate patient selection and preoperative planning remains a crucial part of the evaluation of patients presenting with LIH. A key component remains preoperative computerized tomography imaging of the abdominal wall to evaluate the defect size, contents, the presence of previous mesh, and the relative laxity of the lateral abdominal. Patients presenting with large defects resulting in a weak lateral abdominal wall should be counseled the possibility of having some laxity in the abdominal contour postoperatively. Similarly, those presenting with a lateral abdominal bulge, which is normally seen from denervation of the lateral muscles from previous incision rather than a true hernia, should be counseled on the possibility of similar outcomes. In these cases, the robotic platform may offer better visualization, improved intracorporeal suturing, a more extensive extraperitoneal dissection and the ability to use a large mesh in order to minimize such abnormalities in the contour of the abdominal wall.

One of the critical steps to the procedure is creating an adequate extraperitoneal plane in order to properly seat the mesh with adequate coverage. Prior to this, all efforts should be made to reduce all hernia contents back into the abdomen. An appropriate peritoneal flap starts at about 5 cm from the edge of the defect and extends a minimum of 5 cm in all directions relative to the defect. Keep in mind that as you near the semilunar line, the peritoneum gets thinner and one should take extra care to select the appropriate plane when starting the dissection near this landmark. The same caution should be observed when dissecting close to the ribs and under the diaphragm, where there is less fat between peritoneum and muscle layers. When nearing the hernia sac, efforts should be made to dissect and reduce the sac in order to reveal the true hernia defect.

Primary closure of the defect should be attempted and best done with a V-Loc^TM^ suture allowing the surgeon to bring two fascial edges together by gradually and equally spreading the tension along the entire edge, rather than focusing the tension at the crotch of the suture. When possible, use more than one V-Loc^TM^ along the wound to minimize high tension areas. For larger defects, relaxing maneuvers, such as decreasing the flex position of the patient at the table, which reduces the distance between the ribs and anterior superior iliac spine, and decreasing the pneumoperitoneum can be used to facilitate defect closure.

The choice of mesh remains controversial. We used two polypropelene and two ProGrip^TM^ meshes with adequate results. The ProGrip^TM^ offered the extra advantage of reducing tension along the closed hernia defect. Medium and heavy weight polypropelene offer excellent tensile strength at a minimal cost. The mesh is usually anchored to the anterior abdominal wall using vicryl sutures or evicel. Special care should be taken to avoid sutures far posteriorly, since the lumbar plexus and its branches originate in the intimacy of the psoas muscle. Thus, one should especially avoid traumatic fixation to this area.

Finally, appropriate peritoneal coverage remains a key concept of this repair that requires special attention. Holes in peritoneal coverage should be closed with absorbable sutures to prevent adhesions to intra-abdominal structures. In large peritoneal flap defects that are not amenable to simple closure; alternatives include omental flaps (when available), use of redundant hernia sac, the use of barrier methods such as oxidated cellulose (Surgicel^TM^), or even using coated or absorbable meshes to avoid direct contact between the extraperitoneal mesh and intra-abdominal structures. Adequate overlap, with a full closure or the peritoneum will allow the peritoneal sac to maintain tension of the mesh and avoids dislodgment.

Overall, the TAPP approach avoids a posterior component separation procedure as is required with the extended view total extraperitoneal approach (eTEP). TAPP approach preserves the retromuscular muscle for future repairs, should they become necessary. The reduced cost in disposables, such as coated meshes or tackers, may off load some of the increased cost of using the robotic platform. However, if a patient presents with a defect that is less than 5 cm from the midline or if there is an associated midline defect, an eTEP approach should be strongly considered.

We are continuing to push the limits of minimally invasive hernia surgery. Laparoscopy remains an acceptable, cost-effective, and widely available modality for the repair of many types of hernias, but it does have specific limitations. This is especially true of surgical procedures with tight working spaces that require enhanced dexterity and visualization, much like is required for minimally invasive LIH repairs. The enhanced 3D visualization of the robot, the dexterity and freedom of mobility of the instruments, and the extra robotic hand decrease may overcome the technical difficulties that can be seen with laparoscopy. As early adopters, we expect the robotic platform to become the standard of care for minimally invasive repairs of LIH.

Additional studies are needed to evaluate the relative safety, learning curve, costs, optimal technique, and long-term outcomes of minimally invasive LIH repairs.

## CONCLUSION

In our early experience is evidence that the robotic platform may offer a feasible, lasting, and safe minimally invasive approach to the repair of these notoriously difficult hernias. 
